# Correction: Mutation in the rat interleukin 34 gene impacts macrophage development, homeostasis and inflammation

**DOI:** 10.26508/lsa.202503435

**Published:** 2025-07-16

**Authors:** Stephen Huang, Omkar L Patkar, Sarah Schulze, Dylan Carter-Cusack, Susan Millard, Ginell Ranpura, Emma K Green, Emma Maxwell, Jeeva Kanesarajah, Gary Cowin, Damion Stimson, Nyoman D Kurniawan, Sahar Keshvari, Rachel Allavena, Allison R Pettit, Katharine M Irvine, David A Hume

**Affiliations:** 1 Mater Research Institute-UQ, Translational Research Institute, Brisbane, Australia; 2 National Imaging Facility, Centre for Advanced Imaging, Australian Institute for Bioengineering and Nanotechnology, The University of Queensland , Brisbane, Australia; 3 Centre for Advanced Imaging, Australian Institute for Bioengineering and Nanotechnology, The University of Queensland , Brisbane, Australia; 4 School of Veterinary Science, The University of Queensland , Gatton, Australia; 5 QIMR Berghofer Medical Research Institute, Brisbane, Australia

## Abstract

Characterization of a novel IL34 loss-of-function model reveals overlapping functions of CSF1 and IL34 on CSF1R in the brain and periphery of the rat.

Article: Huang S, Patkar OL, Schulze S, Carter-Cusack D, Millard S, Ranpura G, Green EK, Maxwell E, Kanesarajah J, Cowin G, Stimson D, Kurniawan ND, Keshvari S, Allavena R, Pettit AR, Irvine KM, Hume DA (2025 June 18) Mutation in the rat interleukin 34 gene impacts macrophage development, homeostasis and inflammation. Life Sci Alliance 8(9): e202503264. doi: https://doi.org/10.26508/lsa.202503264. PMID: 40533345.

Following publication, the authors noticed that an old version of Supplementary Figure 2 (Figure S2) had been inadvertently included in this manuscript. The correct version of this figure has now replaced the older version.

**Figure S2.**
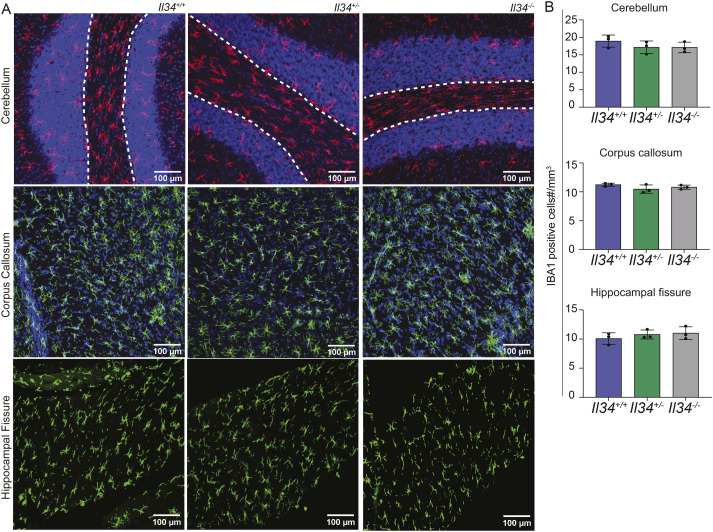
Loss of IL34 does not impact the number of white matter microglia. **(A)** IBA1 (red or green) and DAPI (blue) were stained in brain sections from *Il34*^*+/+*^, *Il34*^*+/−*^ and *Il34*^*−/−*^ rats. Representative images of cerebellum, corpus callosum and the hippocampal fissure are shown. **(B)** Each dot represents counts of microglia per mm^3^ across the different brain regions from independent animals (n = 3). Images were captured on the Olympus FV3000 confocal microscope and scalebars represent 100 μM.

